# Pharmacotherapy for Chronic Idiopathic Constipation and Constipation-Predominant Irritable Bowel Syndrome Beyond Conventional Laxatives: A Narrative Review

**DOI:** 10.7759/cureus.97523

**Published:** 2025-11-22

**Authors:** Saroj K Sahu, Preetam Nath, Bipadabhanjan Mallick, Dibyalochan Praharaj, Suprabhat Giri, Sarat Chandra Panigrahi, Anil C. Anand, Manoj K Sahu

**Affiliations:** 1 Gastroenterology and Hepatology, Kalinga Institute of Medical Sciences, Bhubaneswar, IND

**Keywords:** bile acid modulators, chronic idiopathic constipation, enterokinetic, irritable bowel syndrome with constipation, secretagogues, tenapanor, vibrant capsule

## Abstract

Chronic idiopathic constipation (CIC) and constipation-predominant irritable bowel syndrome (IBS-C) are two highly prevalent functional gastrointestinal disorders that share overlapping features but differ in their pathophysiology and symptom profiles. Both conditions impose a significant socioeconomic burden due to their high prevalence, chronic nature, and impact on daily life. Treatment for chronic constipation begins with lifestyle modifications, including fiber supplementation, and progresses to osmotic and/or stimulant laxatives if the response is inadequate. About half of the patients treated with conventional laxatives do not respond sufficiently, which is due to multiple factors and requires additional therapy. Newer pharmacological agents for CIC and IBS-C are characterized by their targeted mechanisms, including enterokinetics, secretagogues, bile acid modulators, and sodium/hydrogen exchanger-3 inhibitors. Secretagogues like linaclotide improve both motility and sensory symptoms, which are often poorly managed by conventional laxatives. A drug-free treatment called the Vibrant capsule has been approved for CIC, enhancing peristalsis and bowel movements in patients unresponsive to traditional laxatives. Correct differentiation between CIC and IBS-C is essential, as misclassification can lead to suboptimal treatment outcomes. Future therapies are expected to better address the distinct yet overlapping features of these disorders, providing patient-specific treatment, improving symptom relief, and enhancing overall quality of life. The insights synthesized in this review may enhance our understanding of the mechanisms behind pharmacotherapy for chronic constipation and support their application in clinical practice to better patient outcomes.

## Introduction and background

Functional gastrointestinal disorders (FGIDs) constitute a significant global health burden, affecting nearly 40% of the population worldwide and arising from complex disturbances in the bidirectional gut-brain axis. Among these, constipation-predominant FGIDs are especially common. They include chronic idiopathic constipation (CIC), irritable bowel syndrome with constipation (IBS-C), opioid-induced constipation (OIC), and functional defecation disorders (FDDs). CIC and IBS-C are the most common clinical phenotypes in this group. They are marked by chronic constipation symptoms without any structural or biochemical problems. Based on underlying pathophysiological mechanisms and alterations in stool transit and evacuation, CIC can be further subclassified into normal transit constipation (NTC), slow transit constipation (STC), and defecatory disorders (DD) [1].

The worldwide prevalence of CIC is estimated to be around 17%, with a higher incidence noted in women and a gradual rise associated with increasing age [1]. In contrast, the overall prevalence of irritable bowel syndrome (IBS) across all subtypes is about 14.1%, of which IBS-C constitutes roughly 27.9% [2]. IBS-C tends to occur more frequently in younger females [3]. For the diagnosis of chronic constipation (CC), symptoms should be present for at least three months, with onset at least six months before diagnosis, according to established criteria. The key clinical distinction between CIC and IBS-C lies in the presence of recurrent abdominal pain, although a subset of patients with CIC may also experience abdominal discomfort as part of their symptom complex [4].

Both CIC and IBS-C impose considerable social, psychological, and economic burdens on affected individuals and healthcare systems [5]. CIC has been increasingly acknowledged as a factor contributing to frailty, especially in older adults, resulting in considerable deterioration in quality of life and daily functioning [6]. Irrespective of etiology, the overarching therapeutic objectives in constipation-predominant disorders are to relieve symptoms, enhance patient comfort, and re-establish normal bowel function. The aim of this narrative review is to comprehensively summarize current and emerging pharmacological therapies for CIC and IBS-C, with a particular focus on agents that act beyond conventional laxatives, highlighting their mechanisms of action, clinical efficacy, and safety profiles.

Methods

A focused literature search for this narrative review was conducted in PubMed, Embase (OVID), and Google Scholar to identify studies relevant to the pharmacologic and non-pharmacologic management of CIC and IBS-C. Search terms included combinations of “chronic idiopathic constipation”, “IBS-C”, “functional constipation”, “prucalopride”, “lubiprostone”, “linaclotide”, “plecanatide”, “tenapanor”, “elobixibat”, and “vibrating capsule”. Only English-language human studies were incorporated, without any temporal limitations. Eligible study types were clinical trials, observational studies, meta-analyses, guidelines, and mechanistic research. Conference abstracts, non-English articles, preclinical studies, and unavailable full texts were excluded. Reference lists were screened to identify additional publications. Study selection and data extraction were performed independently by multiple reviewers, with disagreements resolved by consensus. As this is a narrative review, no formal risk-of-bias assessment or quantitative synthesis (meta-analysis) was performed.

## Review

Pathophysiology of chronic constipation

Chronic constipation involves multiple overlapping mechanisms rather than a single disease. The underlying mechanisms of CIC and IBS-C are diverse and interconnected, including visceral hypersensitivity, gut-brain dysfunction, neurohormonal pathway dysregulation, microbial dysbiosis, epithelial barrier breakdown, and impaired colonic bile-acid signaling. CIC has three subtypes, with NTC being the most common. NTC features normal colonic transit but sensations of incomplete evacuation due to functional abnormalities, especially pelvic-floor discoordination and psychosocial factors. IBS-C is mainly a pain-focused disorder where constipation occurs alongside recurrent abdominal pain driven by visceral hypersensitivity and gut-brain axis dysregulation [7]. Slow-transit constipation results from intrinsic neuromuscular dysfunction in the colon, such as low-amplitude or uncoordinated contractions, reduced intrinsic nerves and interstitial cells of Cajal, and impaired cholinergic responsiveness, leading to colonic stasis and decreased urge to defecate [8]. Dyssynergic defecation involves uncoordinated rectal propulsion and inappropriate pelvic floor or anal sphincter contraction, often worsened by anatomical abnormalities or altered rectal sensation [9].

Dietary patterns significantly affect IBS-C and CIC development and persistence; insufficient fiber intake, low fluid consumption, and diets high in fermentable carbohydrates can alter stool consistency, slow transit, and increase gas and bloating. Food hypersensitivities and modified nutrient-microbe interactions also affect visceral sensitivity and motility [10]. Psychosocial factors, including stress, anxiety, depression, maladaptive coping, and adverse early life experiences, impact gut-brain signaling by changing autonomic balance, hypothalamic-pituitary-adrenal (HPA) axis activity, and central pain processing, thus worsening constipation and abdominal pain in IBS-C. Bile acid signaling plays a vital role in controlling colonic motility and secretion; disruptions in this pathway contribute to CIC’s pathophysiology. Bile acids activate receptors such as Farnesoid X receptor (FXR) and Takeda G-protein-coupled Receptor 5 (TGR5), which promote chloride and water secretion, stimulate colonic contractions, and modulate sensory pathways. When bile acid delivery to the colon decreases due to increased ileal reabsorption, reduced hepatic synthesis, or altered enterohepatic cycling, these effects are diminished, leading to delayed transit and firmer stools [11].

Conventional laxatives mainly focus on stool consistency or prompting evacuation but do not address the core mechanisms of CIC and IBS-C, such as motility issues, hypersensitivity, secretion impairments, bile-acid signaling alterations, or gut-brain axis dysfunction, resulting in incomplete symptom relief for many. Additionally, standard laxatives often fail to ease abdominal pain, bloating, and the sensation of incomplete evacuation, and their long-term effectiveness, tolerability, and patient satisfaction are suboptimal. This underscores the need for newer, mechanism-based therapies that target fluid secretion, improve motility, reduce visceral hypersensitivity, or rectify neuromuscular and neurohormonal issues.

Treatment goals and stepwise management 

Treatment of chronic constipation aims to increase the frequency of spontaneous bowel movements (SBMs), decrease the severity of abdominal pain or discomfort, and improve overall quality of life (QoL). An SBM is characterized by the absence of laxative use within the preceding 48 hours. When accompanied by a sense of complete evacuation, it is classified as a complete SMB (CSBM).

**Figure 1:**
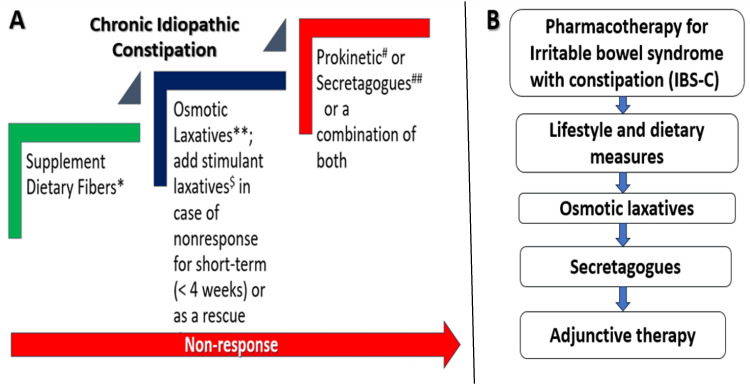
Stepwise management algorithms for chronic idiopathic constipation (A) and constipation-predominant irritable bowel syndrome (B) *phyllium; **polyethylene glycol, lactulose, lactitol; ^$^senna, bisacodyl sodium; ^#^prucalopride; ^## ^lubiprostone, linaclotide/plecenatide, elobixibat, tenapanor IBS-C: constipation-predominant irritable bowel syndrome Image Credit: Saroj Kanta Sahu (Author)

For CIC, treatment should start with increased fiber intake; if the response is inadequate, there should be an addition or switch to an osmotic laxative, ensuring patient involvement in decision-making (Figure 1). Stimulant laxatives can be added as a short-term rescue therapy or as needed if the above measures fail. For patients with an unsatisfactory response, secretagogues, prokinetics, or a combination of secretagogues and prokinetics may be used [12]. Similarly, the treatment of IBS-C begins with dietary and lifestyle changes, followed by the use of osmotic laxatives to manage stool frequency. If there is no improvement, secretagogues (lubiprostone, linaclotide, or plecanatide) should be included. Adjunctive therapies include antispasmodics, low-dose tricyclic antidepressants, or selective serotonin reuptake inhibitors (SSRIs). About 30-50% of patients with CIC or IBS-C fail to respond to lifestyle changes, increased fiber intake, and osmotic laxatives.

First-line pharmacologic management

Bulk-Forming Laxatives (Fiber Supplements)

Bulk laxatives, particularly the soluble fiber psyllium, are potent therapeutic agents for adults with CIC. Psyllium absorbs water and forms a gel, thus enhancing stool frequency and consistency. A regimen of psyllium (up to 20-30 g/day in divided doses with water) is recommended for at least four weeks before deeming it ineffective [13,14]. Patients with more severe NTC or STC generally do not tolerate psyllium well if they experience significant bloating or gas. Psyllium may be considered a first-line option for IBS-C; however, its efficacy is variable, as colonic fermentation can lead to bloating and gas in sensitive patients [15]. In about 50% of patients with CIC or IBS-C, psyllium may not be the ideal solution, necessitating a switch to osmotic laxatives. 

Osmotic Laxatives

Polyethylene glycol (PEG) is one of the safest osmotic laxatives for patients with CIC and IBS-C. Lactulose is less potent than PEG and is often linked to bloating, flatulence, and cramps. Magnesium salts, though effective, carry the risk of hypermagnesemia in specific patient groups. They are safe, affordable, and effective (PEG > lactulose > magnesium salts) and are recommended as the first-line treatment before considering secretagogues or prokinetics [3,12].

Stimulant Laxatives

They act by increasing intestinal motility and fluid secretion in the colon and are best used as short-term rescue therapy for acute constipation [16]. In CIC, where bulk or osmotic laxatives fail, they can be considered for use. Stimulant laxatives such as senna, bisacodyl, and sodium picosulfate may cause abdominal cramping and electrolyte imbalance; hence, they should be used with caution [17].

Second-line pharmacologic management 

Prokinetics

Prucalopride is a selective 5-hydroxytryptamine receptor 4 (5-HT4) agonist [18] (Table 1). It is approved as a second-line treatment for adults with CIC who have failed ≥2 conventional laxatives. The onset of action is within one to two days. The recommended dose is 2 mg daily for adults and 1 mg for the elderly or those with a creatinine clearance < 30 mL/minute (Table 2). Elderly patients have shown higher exposure to prucalopride, which is likely due to impaired renal function; therefore, the dose should be adjusted in the elderly according to renal function [19]. Headache, abdominal pain, nausea, and diarrhea are the most frequently reported adverse events (AEs). The onset of headache is within 2 days of treatment and usually improves over time. Prucalopride is a pregnancy category B1 drug.

**Table 1:** Summary of the mechanism of action and adverse events of novel pharmacological agents for chronic idiopathic constipation and irritable bowel syndrome with constipation. CFTR: cystic fibrosis transmembrane conductance regulator; cGMP: cyclic guanosine monophosphate; CLC2: chloride channel; GC-C: guanylyl cyclase-C receptor; NHE3: sodium–hydrogen exchanger; CIC: chronic idiopathic constipation; IBS-C: constipation-predominant irritable bowel syndrome

Drug	Mechanism of action	Typical onset of action	Indications	Common adverse events
Prucalopride	Highly selective 5-HT4 receptor agonist → increases coordinated colonic peristalsis and accelerates transit.	Within 1–2 days.	CIC in adults	Headache, nausea, abdominal pain, diarrhoea
Lubiprostone	Activates ClC-2 chloride channels on enterocyte apical membrane → increases chloride & water secretion into lumen.	Within 1–2 days.	CIC in both adult males and females; IBS-C only in adult women	Nausea (most common, dose-limiting), diarrhoea
Linaclotide	Guanylate cyclase-C (GC-C) agonist → ↑ intracellular/extracellular cGMP → CFTR activation → chloride, bicarbonate & water secretion; reduces visceral pain signaling.	Stool effect often occurs within ~24 hours	CIC and IBS-C (adults); Functional constipation in children 6-17 years	Diarrhoea, abdominal pain/bloating, flatulence.
Plecanatide	GC-C agonist (uroguanylin analogue) → increases luminal cGMP → CFTR-mediated chloride/water secretion.	Clinical response within days	CIC and IBS-C (adults).	Diarrhoea (most common), abdominal pain, and flatulence.
Elobixibat	Inhibits ileal bile acid transporter (ASBT / IBAT) → decreases ileal bile acid reabsorption → more bile acids reach colon → stimulates secretion & colonic motility.	Often within 24 hours	CIC in adults	Abdominal pain/cramping, and diarrhoea
Tenapanor	NHE3 (sodium/hydrogen exchanger 3) inhibitor on apical enterocyte membrane → reduces intestinal sodium absorption → increases luminal water content and stool transit.	Clinical effects are typically observed within days	IBS-C in adults	Diarrhoea, abdominal pain/discomfort.

**Table 2:** FDA-approved pharmacologic therapies for CIC and IBS-C CIC: chronic idiopathic constipation; eGFR: estimated glomerular filtration rate; IBS-C: constipation-predominant irritable bowel syndrome

FDA-approved drugs	Approved for	Dose	Availability in India	Relationship to meal
Prucalopride	CIC (slow-transit/refractory features)	2 mg once daily (adults) 1 mg per day (severe renal impairment eGFR <30 mL/min/1.73 m² and elderly ≥ 65 years)	Yes	with or without food
Lubiprostone	CIC and IBS-C (especially in women with IBS-C); after osmotic laxative	CIC: 24 mcg twice daily IBS-C (women): 8 mcg twice daily	Yes	After meals with water, to reduce nausea
Plecanatide	For CIC and IBS-C	CIC: 3 mg once daily (oral) IBS-C: 3 mg once daily (oral)	Yes	with or without food
Linaclotide	For CIC and IBS-C, it is often chosen when abdominal pain is prominent	CIC: 145 mcg orally once daily IBS-C: 290 mcg orally once daily Functional constipation (pediatric age group 6-17 years): 72 mcg orally once daily	Yes	at least 30 minutes before the first meal of the day
Tenapanor	IBS-C	IBS-C: 50 mg orally twice daily	No	immediately before the morning and evening meals
Elobixibat^**^	CIC	CIC: 10 mg once daily	Yes	30 minutes before breakfast or dinner

Secretagogues

**Figure 2:**
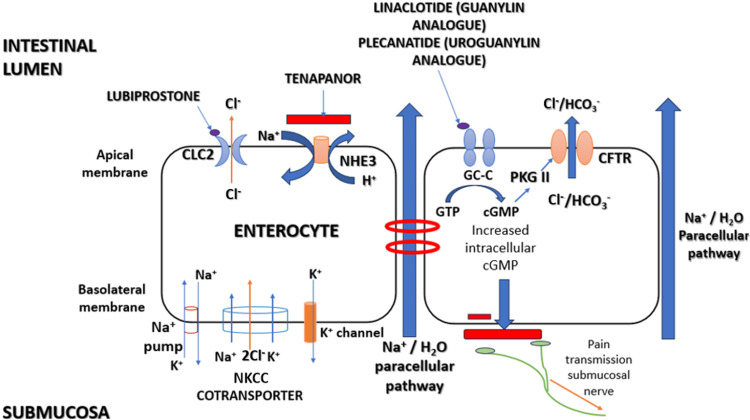
Mechanisms of action of novel pharmacological agents in chronic idiopathic constipation and irritable bowel syndrome with constipation. CFTR: cystic fibrosis transmembrane conductance regulator; cGMP: cyclic guanosine monophosphate; CLC2: chloride channel; GC-C: guanylyl cyclase-C receptor; NKCC: sodium–potassium–2 chloride cotransporter; NHE3: sodium–hydrogen exchanger; PKG II: protein kinase G II. Image Credit: Saroj Kanta Sahu (Author)

Secretagogues are a class of drugs that increase intestinal fluid secretion into the lumen by acting on specific epithelial ion channels or receptors (Figure 2). The extra fluid softens stool, promotes colonic transit, and improves ease of defecation. They are locally acting agents with minimal systemic absorption. They are used as second-line agents for CIC and IBS-C, typically when conventional laxatives fail.

Lubiprostone: Lubiprostone was the first secretagogue to be approved by the United States Food and Drug Administration (FDA) (Table 1). It is a bicyclic fatty acid metabolite analogue of prostaglandin E1 (prostones) [20]. Lubiprostone activates type 2 chloride channels (ClC-2) on enterocytes, increasing chloride and water secretion to accelerate stool transit (Figure 2). The approved dose of lubiprostone for CIC is 24 mcg twice daily. For IBS-C, the approved dose is 8 mcg twice daily, indicated for adult females only. Lubiprostone is prescribed for a 12-week trial to assess response and discontinued if ineffective [21].

In patients with CIC, lubiprostone has been shown to significantly increase the frequency of SBMs without a notable effect on abdominal pain, whereas in IBS-C, it improves both SBM frequency and abdominal pain scores [22,23]. Open-label IBS-C trials showed increased lubiprostone efficacy at 52 weeks [24]. Nausea is the most common side effect, occurring in 20-30% of patients. Taking lubiprostone with food and water reduces the incidence/severity of nausea. Other commonly reported adverse effects are diarrhea, headache, chest discomfort, and peripheral edema. Lubiprostone is a pregnancy category C drug.

Linaclotide: Linaclotide is a GC-C receptor agonist (Table 1). It is an analogue of guanylin, a paracrine hormone, and mimics the action of ST enterotoxin produced by enterotoxigenic *E. coli *(ETEC). The GC-C-cyclic guanosine monophosphate (GC-cGMP) signaling axis is activated by guanylin and uroguanylin in the colorectum and small intestine, respectively. Linaclotide binds to GC-C receptors, increasing both intracellular and extracellular cGMP levels. Intracellular cGMP activates the cystic fibrosis transmembrane conductance regulator (CFTR) channel, leading to accelerated chloride (Cl⁻) and bicarbonate (HCO₃⁻) ion secretion into the intestinal lumen (the GC-C-cGMP-CFTR axis). The movement of Cl⁻ and HCO₃⁻ into the lumen draws sodium (Na⁺) and water osmotically, increasing luminal fluid volume and resulting in softer stool and faster intestinal transit. Extracellular cGMP inhibits colonic nociceptors, thereby relieving pain and improving bowel movement and abdominal discomfort [25].

Lembo et al. published results in 2011 from two large 12-week randomized controlled trials involving 1,276 patients with chronic constipation treated with linaclotide; both doses (145 μg and 290 μg) produced significantly higher CSBM responses than placebo and showed overall superior improvement across all secondary outcomes. Adverse events were similar between groups except for diarrhea, which caused discontinuation in 4.2% of linaclotide-treated patients [26]. Diarrhea, typically occurring within the first one ot two weeks, is the primary adverse effect. A pooled analysis of 2,350 patients with IBS-C revealed that, among those treated with linaclotide 290 µg, over half achieved ≥30% reduction in abdominal pain, discomfort, or bloating within a median of three to four weeks, with a median time to ≥3 CSBMs of four weeks. Late responses also occurred between weeks 4 and 12, and responder profiling indicated that early responders were more often women, White patients, and individuals with less severe baseline abdominal symptoms [27]. Another study on linaclotide reported long-term safety data from a pooled analysis of six randomized controlled trials and two long-term studies involving 3,853 patients treated with linaclotide. Diarrhea was the most common adverse event, usually mild to moderate, with declining rates over follow-up periods up to 104 weeks and no serious diarrhea events, confirming linaclotide’s overall favorable safety profile [28].

Linaclotide is approved for both IBS-C and CIC in adults [29]. It is also the first secretagogue approved for treating functional constipation in children aged 6-17 [30]. Linaclotide is well-tolerated and taken on an empty stomach, 30 minutes before breakfast. The approved dose is higher for IBS-C (290 mcg daily) than for CIC (145 mcg). It is a pregnancy category C drug [31].

Plecanatide: It is the second GC-C agonist approved for treating CIC and IBS-C (Table 1). The production of cGMP activated by GC-C through plecanatide is pH-dependent, whereas that of linaclotide is pH-independent (Table 3). Therefore, linaclotide affects the entire length of the intestinal tract, while plecanatide mainly targets the proximal small intestine.

**Table 3:** Comparative pharmacological and clinical characteristics of linaclotide and plecanatide CIC: chronic idiopathic constipation; IBS-C: constipation-predominant irritable bowel syndrome

Feature	Linaclotide	Plecanatide
Drug Class	Guanylate cyclase-C agonist	Guanylate cyclase-C agonist
FDA-Approved Uses	In adults: Both IBS-C and CIC In the pediatric population (6-17 years): functional constipation	IBS-C and CIC in adults only
Mechanism of Action	Guanylin analogue	Uroguanylin analogue
Onset of Action	3–5 days	24–48 hours
Dosing Instructions	Take on an empty stomach, 30 minutes before a meal	Can be taken anytime, with or without food
Common Side Effects	Diarrhoea, bloating, cramps, nausea	Diarrhoea, bloating, headache, nausea
Pregnancy Category	Category C	Category C

Several phase 3 trials demonstrate that plecanatide effectively increases bowel movement frequency and alleviates GI symptoms in patients with IBS-C and CIC [32-34]. A pooled analysis of two identical phase 3 trials showed that plecanatide 3 mg and 6 mg significantly improved global IBS-C symptoms, with higher overall and sustained responder rates and rapid, lasting improvements in abdominal pain, bloating, fullness, discomfort, and cramping compared with placebo. The drug was well tolerated, with diarrhea as the most common side effect, occurring infrequently and causing few discontinuations. Additionally, there was no clinically significant difference in therapeutic efficacy or adverse events between the 3 mg and 6 mg doses [35].

The recommended dose of plecanatide is 3 mg taken orally once daily. Drug-induced muscle spasms are serious adverse events reported with the use of linaclotide and plecanatide [36]. The link between muscle spasms and plecanatide was stronger than that with linaclotide. Muscle spasms mainly occurred during the early treatment phase, which usually lasts for the first few weeks of therapy, and were more often reported in female patients.

Bile Acid Modulators

Elobixibat: Elobixibat is an inhibitor of the ileal bile acid transporter (IBAT) (Figure 2). Elobixibat was the first IBAT inhibitor approved for the treatment of CIC in Japan (2018). Elobixibat stimulates both motor and secretory functions in the colon. Physiologically, bile acids bind to the highly efficient IBAT and repress hepatic primary bile acid synthesis through the bile acid-FXR-fibroblast growth factor 19 (BA-FXR-FGF19) signaling pathway. Hence, less than 5% of the bile acid pool enters the colon per day [37]. The basis of use of elobixibat in chronic constipation was the observation that patients with diarrhea-predominant IBS (IBS-D) have higher total fecal bile acids, and approximately 15% of patients with IBS-C have low 48-hour fecal bile acids [38].

In the phase 3 trials, elobixibat has been shown to significantly increase the number of SBMs per week compared to placebo in adult patients aged 18-65 with chronic constipation, and an open-label trial lasting up to 52 weeks has shown sustained long-term efficacy [39]. The most common adverse events are mild abdominal pain and diarrhea. 

The secretion of bile acids occurs in response to dietary stimulation, so elobixibat is best taken on an empty stomach, either 20-30 minutes before breakfast or before dinner (Table 2). It is also safe in the elderly [40]. The approved dose is 10 mg once daily.

Sodium/hydrogen Exchanger 3 (NHE3) Inhibitor

Tenapanor: Tenapanor is the first-in-class NHE3 inhibitor. It was first approved in 2019 for the treatment of IBS-C in adults [41]. It inhibits NHE3 in both the intestine and the renal proximal tubule. By inhibiting NHE3, tenapanor reduces sodium absorption in the small intestine and colon, thereby increasing water secretion into the intestinal lumen (Figure 2). This, in turn, accelerates intestinal transit time, resulting in a softer stool consistency in patients with IBS-C. Tenapanor has minimal systemic absorption. Despite minimal systemic absorption, a small degree of systemic NHE3 inhibition, notably in the renal proximal tubule, has been proposed to cause mild metabolic acidosis in some patients.

Tenapanor 50 mg twice daily was studied in two large, randomized, double-blind, phase III IBS-C trials (T3MPO-1: 12 weeks + 4-week withdrawal; T3MPO-2: 26 weeks), both demonstrating significant improvements in abdominal pain, CSBM frequency, and early symptom relief by week one compared with placebo. Diarrhea was the most common adverse event, leading to treatment discontinuation more frequently in the tenapanor group than in the placebo group [42,43]. Like secretagogues, tenapanor is classified as a second-line therapy for IBS-C. The dose is 50 mg orally twice daily before meals. Tenapanor is currently not available for clinical use in India (Table 2). 

Vibrating capsule therapy

**Figure 3:**
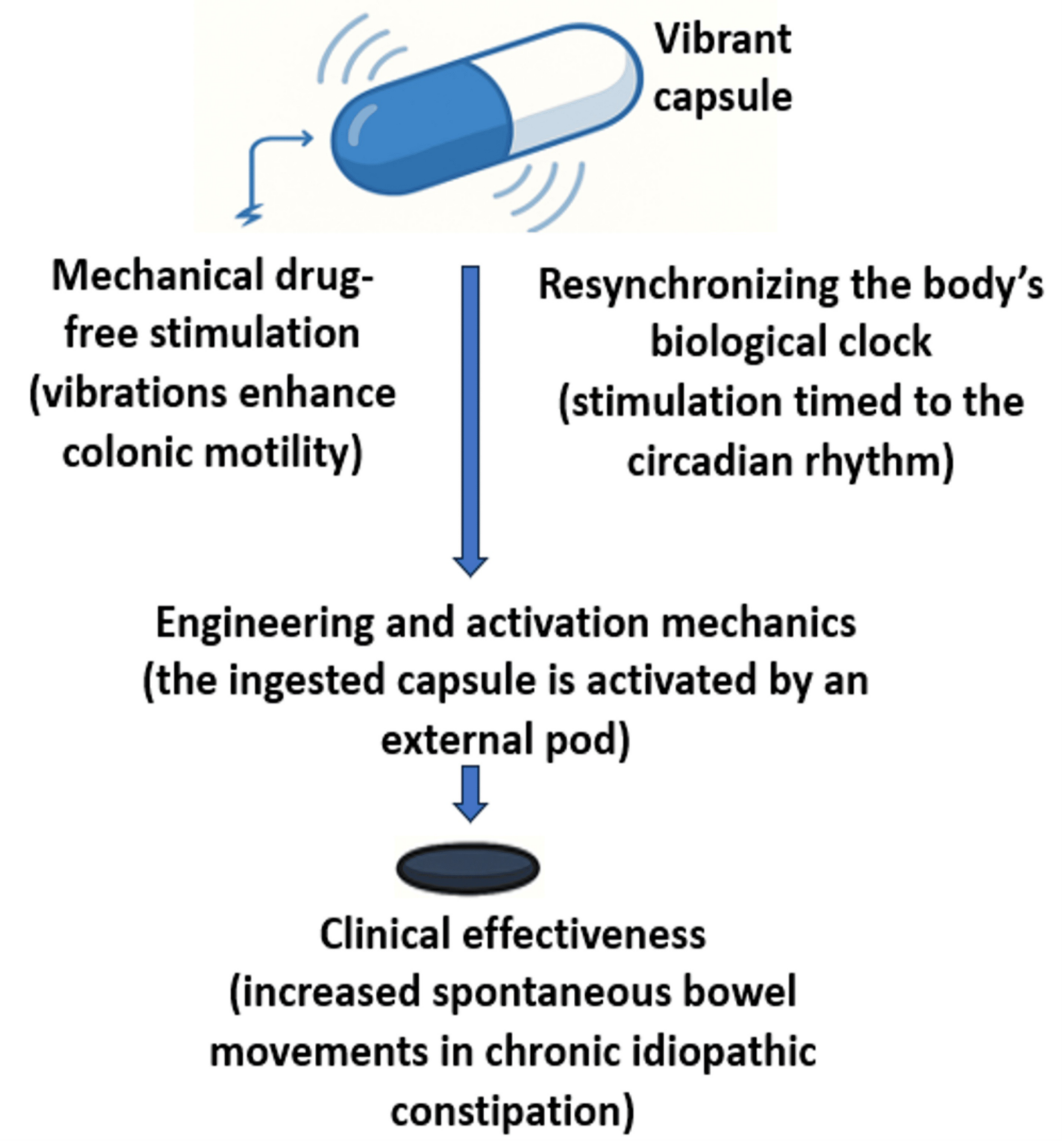
Mechanism of action of the Vibrant capsule system in chronic idiopathic constipation Image Credit: Saroj Kanta Sahu (Author)

Vibrant capsule (VC) is a novel drug-free treatment for CIC. It was approved in 2022 for patients with CIC who have not experienced relief of their bowel symptoms by using laxative therapies at the recommended dosage for ≥1 month. The capsule is taken once a day at bedtime, up to five days a week. The Vibrant system includes a vibrating capsule and a controlling pod. The VC is activated by the control pod using a radiofrequency signal (Figure 3). Swallowed at night, the vibration sequence initiates, which requires a programmed delay of six to eight hours. VC, adjustable via smartphone, promotes defecation by stimulating colonic walls and enhances bowel movements after waking and meals [44].

VC increases the weekly number of complete SBMs in patients with CIC. No serious adverse effects related to treatment were observed, and mild side effects included vibration sensation, diarrhea, and abdominal discomfort [45,46]. Most of these were deemed unrelated to the treatment by researchers. VC may help enhance bowel movements, facilitate defecation, reduce symptoms, and improve QoL in chronic constipation patients. The vast majority of patients (83%) found the system convenient, with an overall satisfaction rate of 71% [47,48].

Future perspectives

Naronapride is an investigational new drug for CIC. It has dual action, acting as a serotonin 5-HT4 receptor agonist and a dopamine D₂ receptor antagonist [49]. It is a locally acting pan-GI prokinetic. Apart from CIC, the drug is also in trials for gastroparesis and proton-pump inhibitor (PPI)-nonresponsive symptomatic gastroesophageal reflux disease (GERD) [50]. Drugs like tenapanor and elobixibat are likely to gain wider adoption beyond their initial markets. Clinical trials combining prokinetics and secretagogues or secretagogues and NHE3 inhibitors may be expected in future clinical trials. More mechanistic diversity may be addressed in future trials aimed at alleviating pain, bloating, barrier dysfunction, and gut-brain signalling. The future of pharmacotherapy for CIC and IBS-C is moving toward targeted, multimodal, and personalized approaches with tenapanor, bile acid modulators, and new-generation 5-HT4 agonists (e.g., naronapride) at the forefront, complemented by microbiome-based and neuromodulatory strategies.

Limitations

This article presents a narrative review and does not adhere to a systematic search strategy or protocol. As such, the selection of studies may not be comprehensive, and there is potential for selection bias in the literature discussed. The perspectives presented are intended to provide an overview of current trends and future directions in the pharmacotherapy of CIC and IBS-C, rather than an exhaustive evidence synthesis.

## Conclusions

Current therapeutic options for CIC and IBS-C, including prucalopride, lubiprostone, secretagogues (linaclotide and plecanatide), tenapanor, elobixibat, and VC, provide substantial improvements in stool frequency, consistency, and overall symptom burden. Their relative effectiveness differs based on the mechanistic targets. Prucalopride is most effective in slow-transit or refractory constipation; lubiprostone and secretagogues enhance secretion and often reduce abdominal pain; tenapanor modulates sodium transport and visceral hypersensitivity in IBS-C; and elobixibat (approved in Japan) accelerates colonic transit through bile acid stimulation.

Linaclotide had the strongest and most consistent reduction in abdominal pain across IBS-C trials, with the highest pain responder rates among approved agents. VC offers a non-pharmacologic option for select patients but requires further evidence. Safety considerations include nausea with lubiprostone, diarrhea and cramping with secretagogues, tenapanor, and elobixibat, as well as special-population dosing concerns and potential cardiovascular risks with prokinetics. Future research should emphasize robust head-to-head comparisons, long-term safety data, real-world effectiveness, biomarkers for predicting responders, combination approaches, cost-effectiveness studies, and the incorporation of multi-omics and machine-learning methods to individualize treatment.
